# Post-Pandemic Shifts in Sustainable Food Behavior: A Systematic Review of Emerging Consumer Trends

**DOI:** 10.3390/nu17233737

**Published:** 2025-11-28

**Authors:** Maria P. Koliou, Dimitris Skalkos

**Affiliations:** Laboratory of Food Chemistry, Department of Chemistry, University of Ioannina, 45110 Ioannina, Greece

**Keywords:** Hunger (H), Food Choice Motives (FCM), Food Responsiveness (FR), Emotional Over Eating (EOE), Emotional Under Eating (EUE), Enjoyment of Food (EF), Satiety Responsiveness (SR), Food Fussiness (FF), Slowness in Eating (SE)

## Abstract

The COVID-19 pandemic and its associated economic stressors have profoundly reshaped consumer eating behaviors, presenting an urgent and underexplored challenge for the academic community. This interdisciplinary review critically examines how these disruptions have influenced both food approach and food avoidance patterns, offering a structured analysis of eight key behavioral parameters: Hunger (H), Food Responsiveness (FR), Emotional Overeating (EOE), Enjoyment of Food (EF), Satiety Responsiveness (SR), Emotional Under Eating (EUE), Food Fussiness (FF), and Slowness in Eating (SE). Drawing on recent literature, we highlight significant shifts in these traits—such as heightened hedonic hunger, age-related changes in food preferences, and gender-specific emotional-satiety dynamics—underscoring the complex interplay between emotional states, physiological cues, and behavioral tendencies. Grounded in the systematic examination of peer-reviewed studies in the post-COVID period, this review offers a robust and comprehensive synthesis of current evidence. The novelty of this work lies in its integration of findings into targeted proposition statements for each parameter, visually supported by original flow charts. These culminate in the development of a “Consumers’ Eating Behavior Index”—a conceptual tool designed to guide researchers, healthcare professionals, and policymakers in understanding and responding to post-pandemic dietary transformations. By emphasizing the emotional and psychological dimensions of eating, this index offers a timely framework for designing tailored public health interventions that promote sustainable nutritional habits. This study calls for renewed academic attention to the behavioral consequences of global crises, positioning eating behavior research as a critical frontier in post-COVID recovery and resilience.

## 1. Introduction

During a crisis, such as COVID-19 and economic instability, eating habits may shift due to stress, leading to overeating or loss of appetite [1]. Afterwards, some maintain those habits while others revert to normal, influenced by economic factors. Eating habits are shaped by food choice motives, such as taste and health, as well as food behaviors, including eating behaviors and consumption patterns [2]. We have systematically reviewed the effects of the COVID-19 pandemic on food choice motives (FCM) and noted a marked rise in online shopping, price hikes, and more conservative approaches to buying quality foods [3]. In contrast, familiarity, convenience, and sensory appeal showed little change, while mood and stress motives increased food consumption dangerously [4]. These two factors also determine sustainable eating behavior [5].

The distinction between food choice and food behavior primarily hinges on the underlying factors and the breadth of their influence. Food choice refers to the decision-making processes regarding which foods to consume, influenced by a variety of factors [1,3]. This involves conscious decisions about what foods are preferable or desirable at a given time.

### 1.1. Food Behavior and Appetitive Traits

Food behavior encompasses the actions and decisions individuals make regarding food consumption, influenced by various psychological, social, and environmental factors [1,3]. It involves observable patterns and habits, such as meal frequency, food choices, and eating contexts, which can vary according to life circumstances [6]. Food behavior and appetitive traits, while related, are distinct concepts. Appetitive traits can influence food behavior, but they do not encompass the entire spectrum of eating actions and decisions. Understanding the distinction between the two is important for research in nutrition, psychology, and public health [7,8]. Appetitive traits are individual characteristics that influence a person’s tendency and motivation to eat [9,10]. These traits are more innate and stable over time compared to the more flexible nature of food behavior. Appetitive traits often underpin food behavior. For example, an individual with high food responsiveness (FR) may exhibit sustainable food behaviors such as frequent snacking or overeating in response to food cues [11]. Conversely, sustainable food behavior can also influence the expression of appetitive traits over time [12].

Most research into food behavior- or appetite traits employs validated and reliable questionnaires. Over the past fifteen years, numerous questionnaires have been widely used to assess appetite traits comprehensively. Notably, the ‘Three Factor Eating Questionnaire’ (TFEQ) [13] and the ‘Dutch Eating Behavior Questionnaire’ (DEBQ) [14] are common tools for adults, while the Child Eating Behavior Questionnaire (CEBQ) is widely used for children [15,16]. The Baby Eating Behavior Questionnaire (BEBQ) measures eating behaviors in infants and was developed as part of a broader study on appetitive traits [17]. Completing the set of life-stage-appropriate tools is the Adult Eating Behavior Questionnaire (AEBQ), which assesses various dimensions of eating behavior in adults, further extending the utility of standardized questionnaires across age groups [18].

These questionnaires serve as essential tools for researchers and clinicians, enabling comprehensive assessment and targeted interventions for sustainable eating behaviors across various life stages. From infancy to adulthood, each questionnaire offers structured insights into cognitive, emotional, and environmental influences on eating habits. By identifying patterns like emotional eating and restraint, these tools facilitate tailored strategies for managing obesity, eating disorders, and promoting healthy dietary practices. turned structured approach supports evidence-based research and clinical practices, ultimately aiming to enhance overall health outcomes and quality of life across diverse age groups.

From the above, it is clear that the methodology of questionnaires circumvents the high costs and logistical challenges associated with laboratory and neural measurements of eating behavior, facilitating broader and more efficient data collection. Utilizing these questionnaires enables researchers to gather extensive data across large populations without the financial and technical barriers that come with more complex measurement techniques [19]. The ability and validity of these questionnaires ensure that the data collected is both accurate and meaningful, providing a robust foundation for understanding eating behavior on a large scale. Consequently, this approach has become the preferred method in the field, offering a practical and cost-effective alternative to traditional laboratory-based studies. By leveraging these tools, researchers can explore food behavior in diverse populations, leading to more comprehensive and generalizable findings. This strategy, as highlighted by Carnell, has significantly advanced our ability to study related behaviors and their underlying mechanisms, making substantial contributions to the field of sustainable nutritional science and behavioral research [20].

### 1.2. Review’s Objectives

This review seeks to contribute to our understanding of how pandemic and eco-nomic distress shaped eating behaviors, with implications for public health strategies, interventions, and policy development aimed at promoting sustainable well-being in the post-pandemic era. Through this analysis, we strive to provide valuable insights for researchers, policymakers, and healthcare professionals navigating the challenges posed by the COVID-19 pandemic and its aftermath on dietary behaviors and nutritional health.

In this paper, we undertake a review of reported data concerning the eight main parameters that constitute eating behaviors.

“Food Approach” behaviors:

- Hunger (H);
- Food Responsiveness (FR);
- Emotional Overeating (EOE);
- Enjoyment of Food (EF).

“Food Avoidance” behaviors:

- Satiety Responsiveness (SR);
- Emotional Under Eating (EUE);
- Food Fussiness (FF);
- Slowness in Eating (SE).

For each parameter, after the review of the reported data, we come up with a proposition summarizing the overall findings.

## 2. Research Approach and Review of Relevant Literature

### 2.1. Research Approach: This Subsection Outlines the Methodological Framework and Search Strategy Applied in Conducting the Review

**Figure 1:**
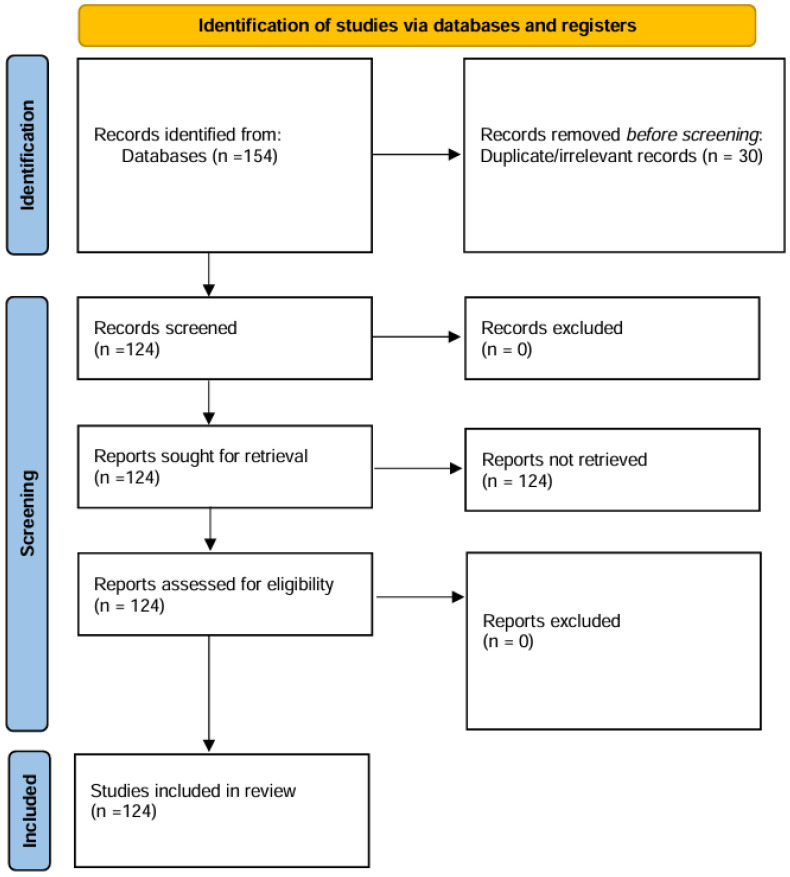
PRISMA 2020 flow diagram of study selection.

This review followed PRISMA-ScR guidelines for scoping reviews and aligned with the JBI Manual for Evidence Synthesis, based on the methodological guide by Arksey and O’Malley [21]. The study selection process is illustrated in Figure 1, following PRISMA 2020 guidelines.

We adhere to the methodology employed in our prior review on FCM [3], ensuring consistency and reliability in our approach. By maintaining the same rigorous process, we uphold standards of accuracy and comparability in data collection, analysis, and interpretation. This methodology enables us to build upon existing knowledge, validate findings, and contribute to cumulative research efforts effectively. By aligning with established practices, we ensure our study’s credibility and relevance, fostering a cohesive and informed understanding of the subject matter. This approach supports robust conclusions and facilitates meaningful contributions to the academic and scientific community.

A search was conducted on PubMed, Google Scholar, and Science Direct for studies published between 2020 and 2025, including early-access and in-press articles from the current year, employing predefined search terms to ensure the integration of the most recent and relevant findings. Additionally, studies published before this period were included in the review, only to give the required knowledge background of each subject matter. Initially, the search terms were employed across these three databases to grasp the current research landscape. Subsequently, alternative phrasing searches were conducted, and guidance on search strategy was refined. Finalization included incorporating 8 specific search terms (research themes) for this systematic review, ensuring comprehensive coverage of relevant literature.

### 2.2. Protocol and Registration: This Subsection Clarifies the Registration Status of the Review Protocol and Its Alignment with Established Guidelines

No formal registration of the review protocol was undertaken. Nevertheless, the review was conducted in accordance with the PRISMA-ScR guidelines and the JBI Manual for Evidence Synthesis to ensure transparency, reproducibility, and methodological rigor.

### 2.3. Eligibility Criteria and Limitations: This Subsection Presents the Inclusion and Exclusion Criteria Applied During Study Selection, as Well as the Methodological Limitations Encountered

This search took place in February and March of 2025. No restrictions or filters were applied to prevent the exclusion of potentially relevant papers. Eligibility assessment was conducted based on title, abstract, and full text. Two researchers (DS and MPK) independently screened articles for eligibility, adhering to predefined inclusion criteria. This rigorous process is aimed at ensuring thorough coverage and unbiased selection of studies relevant to the research objectives.

▪ Limitation to papers published in the years 2020, 2021, 2022, 2023, 2024 (including prior papers for the definition of terminologies).
▪ Studies investigating the connection of the post-COVID era and eating behavior (in some cases defined as appetite traits).
▪ Studies in English only.

1. The search was comprehensive to encompass all relevant studies aligned with the review’s aim. No authors were contacted for additional information, maintaining the review’s independence and reliance solely on available literature for data extraction and analysis.
2. The limitations of the review process included the following factors:
  ▪ The review only considered full-text publications in English, potentially introducing selection bias due to language limitations.
  ▪ Dietary intake was evaluated through self-reported data, a common method in nutritional research, susceptible to misreporting or underreporting, potentially affecting study outcomes.
  ▪ Due to the predominance of cross-sectional studies, self-reporting dietary data (through questionnaires) assessing bias and study quality was challenging within this review due to inherent limitations in study design and variations in methodological rigor among included studies.

Quality assessment compared to longer-term cohort or cause-effect research was unfeasible due to the predominance of cross-sectional studies in the review.

## 3. Results

**Figure 2:**
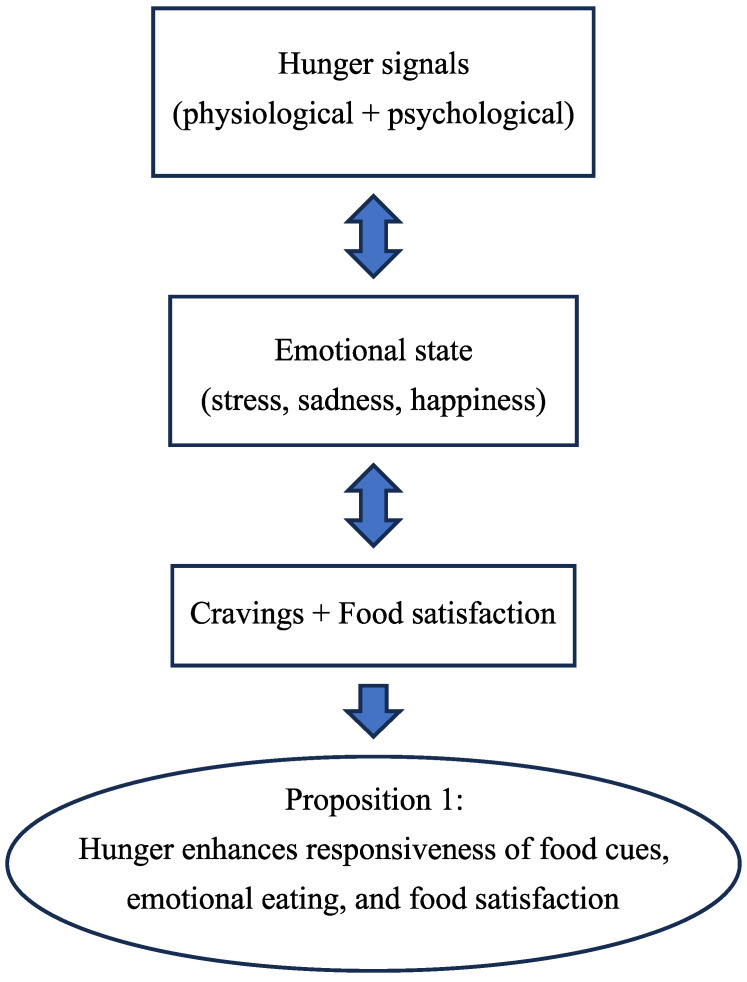
Flow Chart 1—Hunger (H). Conceptual flow chart summarizing the influence of hunger on food cues, emotional eating, and food satisfaction in the post-COVID era.

**Figure 3:**
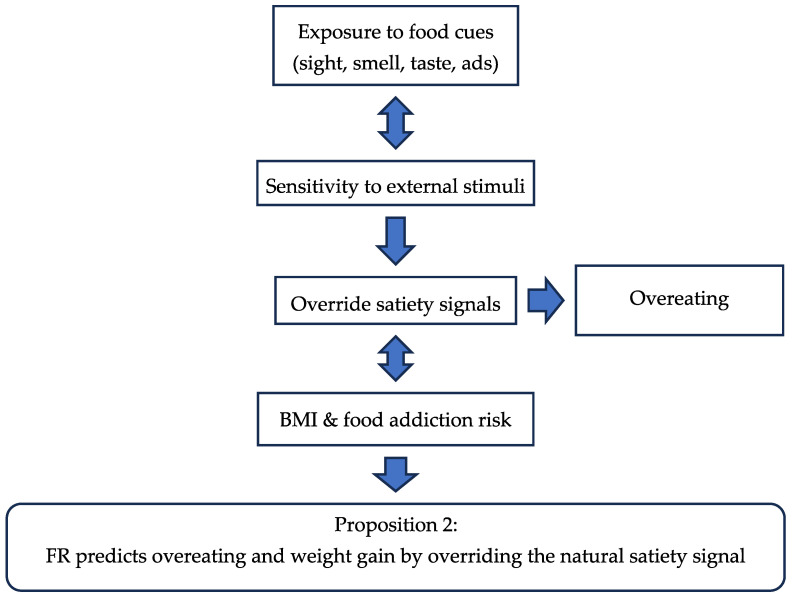
Flow Chart 2—Food Responsiveness (FR). Conceptual flow chart illustrates the role of food responsiveness in cue-driven eating, BMI, and satisfaction with food-related life.

**Figure 4:**
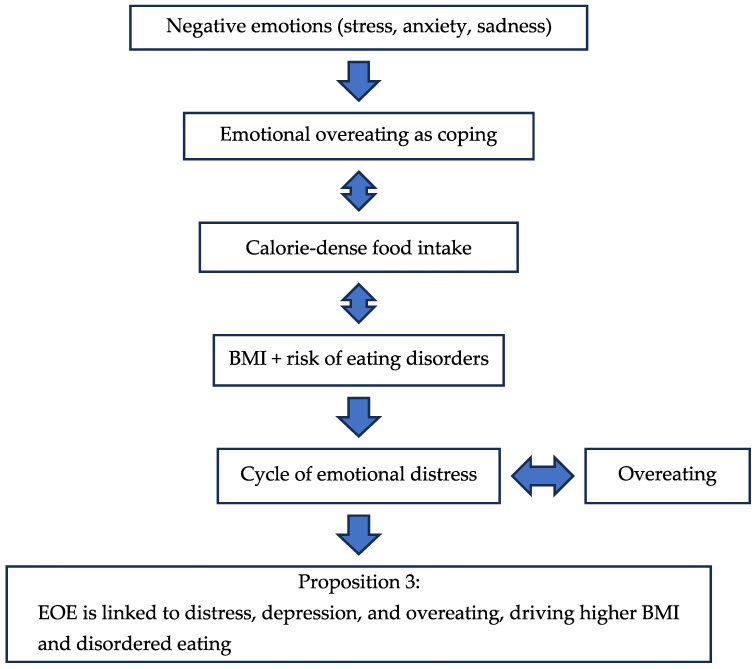
Flow Chart 3—Emotional Overeating (EOE). Conceptual flow chart depicting the impact of emotional overeating on obesity risk and compulsive eating behaviors.

**Figure 5:**
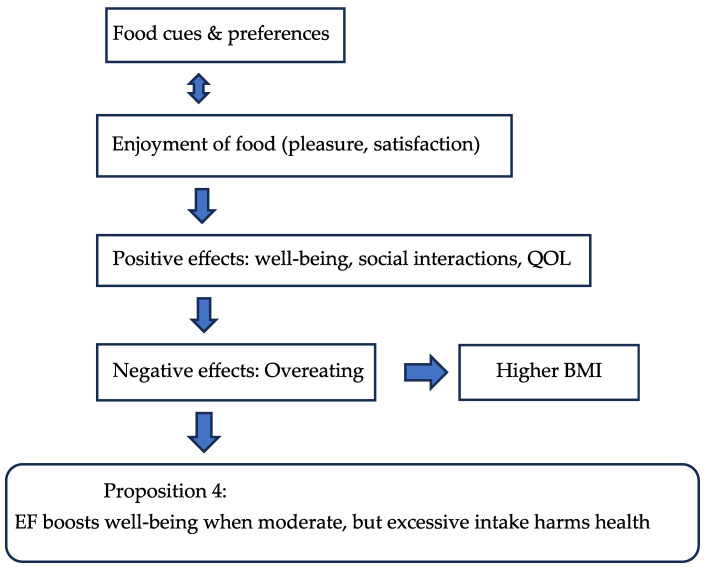
Flow Chart 4—Enjoyment of Food (EF). Conceptual flow chart highlighting the Moderating role of food enjoyment on hunger, satisfaction, and sustainable eating choices.

**Figure 6:**
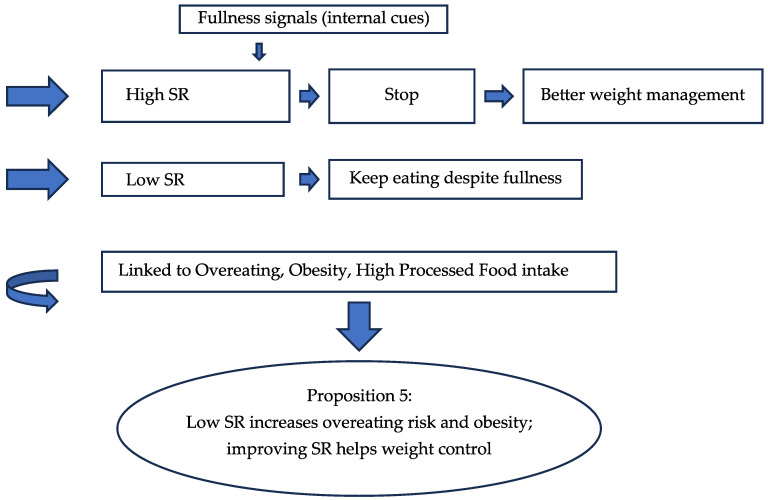
Flow Chart 5—Satiety Responsiveness (SR). Conceptual flow chart showing the protective role of satiety responsiveness against overeating and obesity.

**Figure 7:**
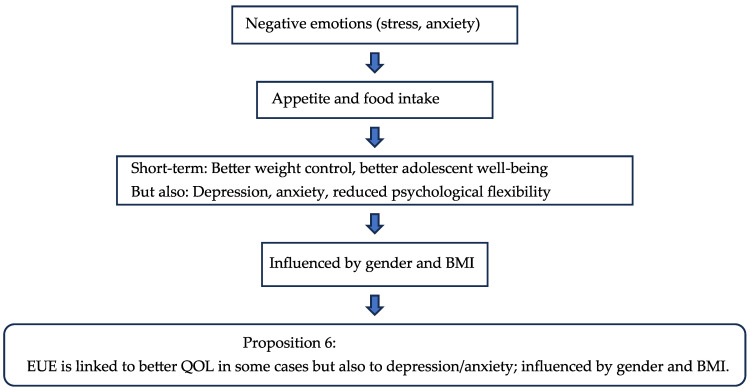
Flow Chart 6—Emotional Undereating (EUE). Conceptual flow chart summarizing the maladaptive coping mechanisms of emotional undereating and their impact on intake regulation.

**Figure 8:**
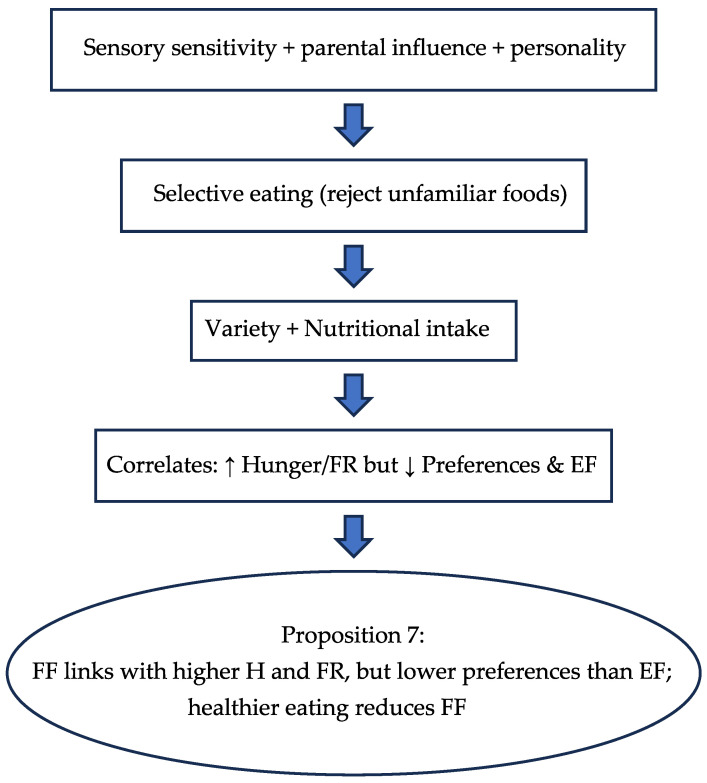
Flow Chart 7—Food Fussiness (FF). Conceptual flow chart illustrating the limiting effect of food fussiness on dietary variety and sustainability (arrow ↑ indicates increase/arow ↓ indicates decrease).

**Figure 9:**
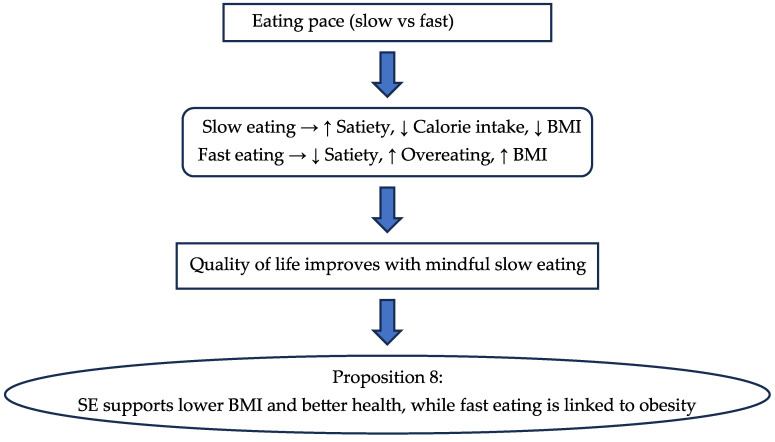
Flow Chart 8—Slowness in Eating (SE). Conceptual flow chart shows the positive influence of slow eating on satiety, healthier intake, and sustainable food behavior (arrow ↑ indicates increase/arow ↓ indicates decrease).

Following a systematic review of all relevant studies, we examined shifts in consumers’ eating behavior in the post-COVID era as shown in Table 1, Table 2, Table 3, Table 4, Table 5, Table 6, Table 7 and Table 8. The key findings for each of the eight parameters are presented through Figure 1, Figure 2, Figure 3, Figure 4, Figure 5, Figure 6, Figure 7, Figure 8 and Figure 9, which summarize the main influencing factors and outcomes identified in the literature. Each chart concludes with a proposition that encapsulates the overall evidence for the respective parameters. This is indeed the main novelty of our work. In addition, Table 9 provides a Conceptual Correlation Table of Eating Behavior Parameters, while Figure 9 illustrates the conceptual relationships among the eight parameters. This analysis encompassed behaviors related to food approach and food avoidance, highlighting how the pandemic influenced dietary choices and preferences.

The terms “appetite,” “stressors,” “hedonic hunger,” “etiology,” “body mass index,” “preference,” “desire,” “emotional state,” “overweight”, etc., were classified under the category of sub-domains because they represent distinct yet interrelated conceptual elements that are fundamental to understanding the complex dynamics of food behaviors, body weight regulation, and psychological influences on food intake. Each of these factors contributes a unique aspect to the multifaceted processes that impact appetite control, weight management, and associated behavioral outcomes.

By grouping these factors as sub-domains, a more nuanced analytical framework is established, allowing for the in-depth exploration of each term’s role within the broad-er context of food behaviors. This classification also highlights the interconnectedness of these elements, as each sub-domain may be influenced by biological, psychological, or environmental factors to varying degrees. Through this approach, the study is better positioned to dissect the individual contributions and interactions of each sub-domain.

### 3.1. Food Approach Behaviors

#### 3.1.1. Hunger (H)

Hunger is the physiological and psychological state driving the pursuit and consumption of food. This concept involves internal signals indicating nutritional need and external actions to satisfy this need. Lowe and Butryn describe hunger as a homeostatic drive for energy balance [22], while Berthoud emphasizes the role of brain mechanisms in regulating hunger and guiding food-seeking behaviors [23]. Thus, hunger is a complex interplay of bodily signals and behaviors aimed at obtaining food to maintain energy homeostasis.

An example is the case of university students in the United States with moderate to severe anxiety exhibited elevated hunger levels, tendencies for emotional eating, heightened responsiveness to food cues (FCR) and feelings of fullness, and diminished pleasure in eating [6]. College students who prioritize internal hunger and fullness cues over external factors tend to regulate their food intake effectively, leading to healthier weight management compared to those influenced by external surroundings [24].

Our findings show that in the post-COVID era, while hunger affects health perceptions differently for those who practice restrained eating, it does not change how they rate food tastiness [25]. Increased hunger, however, significantly boosts satisfaction with food-related life [26], indicating that greater hunger leads to higher appreciation and enjoyment of meals. This underscores the influence of physiological states on food satisfaction.

Hunger significantly affects food cravings, which are also influenced by one’s emotional state [27]. Increased hunger heightens food desires, but these cravings are shaped by emotions such as stress, sadness, or happiness [28]. This interaction between hunger and emotions demonstrates the complex relationship between physiological needs and psychological factors in determining food cravings.

Emerging research in the post-COVID era has increasingly highlighted the intricate ways in which hunger is no longer solely governed by physiological cues but is significantly modulated by emotional states and hedonic stimuli [29]. Studies indicate that individuals are more susceptible to experiencing hunger in response to emotional fluctuations—such as stress, anxiety, or even boredom—rather than purely biological need [30]. Moreover, the presence of hedonic triggers, including the sight, smell, and anticipated pleasure of food, has been shown to amplify hunger sensations, particularly in environments saturated with palatable and energy-dense options [31]. This shift underscores a growing recognition that post-pandemic hunger is deeply intertwined with psychological and sensory factors, reflecting a broader transformation in how individuals perceive and respond to internal and external food-related cues [30,32].

Hunger has not been linked to negative psychological states in the new era like sadness, anxiety, or anger. The findings suggest that hunger interacts more with positive emotions, showing a mutual influence between them, but does not significantly affect or get affected by negative emotions [33]. Additionally, behaviors resembling food addiction may not directly correlate with higher BMI or obesity [34].

Food cues significantly heightened hunger more than neutral or stress-related stimuli, regardless of weight status [27]. Hungry participants had difficulty distinguishing emotional reactions from neutral stimuli due to increased arousal, and they also demonstrated better memory for food-related stimuli.

Food cravings were influenced by hunger and stress, with hunger also predicting loneliness in the post-COVID period [25]. Increased hunger correlated with higher satisfaction in food-related life, demonstrating that stronger hunger enhances food satisfaction.

Hunger can worsen emotional stress, especially in older males, affecting mood and well-being today. Emotional strain amplifies hunger signals, highlighting the importance of addressing both nutritional needs and emotional health, particularly with age. Murakami et al. [35] found that older adults showed less emphasis on convenience and sensory appeal in food choices in the new era, while they valued tradition and safety more, and tended to eat more slowly, reflecting different dietary priorities compared to younger individuals [36].

Pregnant women often eat in the absence of hunger (EAH), consuming both highly and minimally processed foods, which is associated with addictive-like behaviors but not hedonic hunger [37]. Impulsivity does not influence this relationship. The DeAguiar and Seo study has shown that focusing on physical hunger and internal hunger and fullness signals mediates the link between interoceptive accuracy and eating disorder risk [38].

In the post-COVID period individuals with overweight or obese place less importance on “health,” “natural concerns,” and “Hunger” in their food choices compared to those with normal weight [39]. Conversely, those with normal weight value health, natural concerns, and hunger more, indicating that food motivations shift with body weight. The findings show that individuals who feel hunger and food cravings are more likely to overeat, especially if they have low satiety responsiveness (SR) [40]. Those who do not easily feel full are more susceptible to hunger and cravings, leading to higher food consumption. This highlights how hunger sensitivity and cravings contribute to overeating, particularly in those who struggle to recognize fullness cues.

Research in the new era after COVID indicates that higher levels of hunger are associated with stronger food preferences, suggesting that hunger significantly influences food choices [40,41]. Additionally, intermittent fasting increases hunger and decreases light physical activity on fasting days compared to non-fasting days, although it does not significantly alter food cravings or daily calorie intake [42,43].

Beyond the body’s physiological need for food, there’s another type of hunger known as hedonic hunger [22]. This form of hunger is driven by external factors, such as the sight or smell of food, and can occur even when the body doesn’t need more calories for energy [28]. Hedonic hunger is more prevalent in women and tends to decrease with age as shown by recent findings [44]. Hedonic hunger, driven by external cues rather than a need for energy, is linked to higher BMI, reduced physical activity, night-time snacking, dieting, and in-creased food cravings [45].

Recent findings in post-pandemic nutritional psychology have revealed that hedonic hunger—a form of appetite driven by the pursuit of pleasure rather than physiological need—continues to be notably elevated among younger individuals and women [32]. This heightened sensitivity to external food cues, such as the sight, smell, or imagined taste of palatable foods, reflects a shift in eating motivations that prioritize sensory gratification over energy balance [45]. In these populations, hedonic hunger is not only more prevalent but also closely associated with psychological traits such as impulsivity, which can lead to spontaneous and unregulated eating behaviors. Moreover, this pattern of pleasure-driven consumption has been linked to symptoms resembling food addiction, where individuals experience compulsive cravings and a diminished ability to resist highly rewarding foods, despite the absence of true hunger [46]. These tendencies suggest that youth and women may be particularly vulnerable to the reinforcing cycle of emotional eating and reward-seeking, which can contribute to challenges in maintaining healthy dietary habits and managing body weight in the post-COVID era. It also correlates with impulsive behaviors and lower self-esteem, reflecting the influence of both physical and psychological factors on eating habits [23,47].

Today, factors like lack of physical activity, nighttime snacking, and diet non-adherence increase hedonic hunger, driving cravings for pleasure rather than need [45]. Despite higher body weight, older adults often have reduced hunger awareness, emotional eating, and food enjoyment compared to younger individuals today [35,48].

In addition, today, hunger doesn’t universally lower perceived food quality but makes individuals rate utilitarian foods lower compared to hedonic foods [49]. In contrast, satiated individuals don’t make this distinction, showing hunger’s selective impact on evaluating food based on utility versus pleasure [25].

Recent studies, after the COVID epidemic, indicate that heightened sensitivity to food cues and diminished responsiveness to feelings of fullness are linked to increased overeating and overweight/obesity both at present and over time [50]. Findings propose that these two aspects—reactivity to food cues and ability to recognize satiety—may lie on a shared spectrum and represent viable targets for interventions aimed at controlling overconsumption and promoting weight loss [51].

Today, in the new era, the elevated hedonic hunger increases the likelihood of choosing sweet, starchy, and fast foods, prioritizing taste over nutrition and contributing to obesity [52]. Women may overeat palatable foods, often valuing food enjoyment over physiological hunger cues [53]. Research shows a strong link between BMI and hedonic hunger, with women experiencing higher levels than men [54]. Hedonic hunger tends to decrease with age but is associated with increased BMI, especially in women. As hedonic hunger increases in overweight adults, self-esteem tends to decrease, and self-stigmatization about weight rises, leading to greater feelings of shame and lower self-worth [40]. Hedonic hunger is linked to brain network structures, influencing self-control in eating among older adults, shaped by personal and external factors [55]. Among young adults, hedonic hunger is prevalent, associated with food addiction, and correlates with obesity [52].

**Table 1:** Papers included in this review are categorized under the theme ‘Hunger’ and its related sub-themes.

Eating Behavior Discource	Sub-Domains	Paper Declaration Number
Hunger (H)	Appetite	[6,13,22,28,29,42,43,50,56,57,58,59,60,61,62]
	Stressors	[4,24,31,33,41,63,64,65,66,67,68,69,70]
	Hedonic Hunger	[22,23,28,30,32,40,44,45,46,47,49,52,71,72,73,74,75,76,77]
	Etiology	[29,38,50,55,57,59,61,62,78]
	Body Mass Index	[32,34,35,36,40,45,51,61,64,68,79,80,81,82,83]

Controlled test meals show that hunger increases the willingness to try unfamiliar foods, highlighting its role in food intake regulation [56]. Preparing food actively raises both hunger and motivation to eat, while passive preparation boosts motivation without affecting hunger. Distractions had little impact on hunger or eating motivation [84]. The papers evaluated above for “hunger” are presented in Table 1. Flow chart 1 shows the influencing factors for hunger which lead to the proposition for the respective parameter for the post-COVID period.

#### 3.1.2. Food Responsiveness (FR)

FR, a key aspect of food approach behavior, refers to an individual’s sensitivity and reaction to food-related stimuli, such as sight, smell, and taste, driving the motivation to eat. Carnell and Wardle link heightened food responsiveness to increased food intake and higher BMI [20]. Schüz et al. associate it with frequent cravings and emotional eating, influenced by psychological and environmental factors [85]. Understanding this concept aids in developing strategies to mitigate overeating and promote healthy eating behaviors.

Food cue responsiveness (FCR) today refers to how individuals react to external food-related cues—like advertisements, smells, or visuals—triggering appetite even when they aren’t physically hungry [86]. In the new era, this heightened responsiveness can lead to over-eating, as these cues may prompt eating beyond actual energy needs, contributing to excess calorie intake and obesity in both youth and adults [10,39]. In the post-COVID period, FCR override natural hunger and fullness signals, making weight management challenging, especially in environments rich with food marketing, thus reinforcing unhealthy eating habits [11,87,88]. Food cue reactivity has intensified in digital environments [89], overriding satiety signals, and further complicating efforts to regulate intake and maintain healthy eating patterns [90,91].

FR proves to be today a key factor in shaping eating habits, which are vital to adolescents’ overall well-being and quality of life [92,93]. As people age, their food preferences shift, with older adults often prioritizing traditional, organic, and safe foods over options chosen for convenience or sensory pleasure [26,48]. This change reflects evolving lifestyle priorities and health concerns that come with aging.

Studies before COVID show that higher food preference scores are positively linked to increased FR [7,8,9]. Individuals with higher scores experience stronger H, greater EF, and are less picky, though they tend to eat less when emotional [18,71]. This suggests that those more responsive to various foods feel hungrier and gain more pleasure from eating [72,79].

FR, among others, was found to be a significant predictor of satisfaction today with food-related life, with correlation values of 0.38 [10]. An increase in this factor has been associated with greater satisfaction in food-related aspects of life [8,39]. This indicates that people who derive more pleasure from eating, respond more to food cues, and experience hunger more intensely tend to be more satisfied with their food experiences [11].

FR doesn’t significantly predict changes in the consumption of high-energy-dense (HED) sweet and savory foods [79]. Factors like sensitivity to food cues, EF, emotional eating, and recognizing fullness did not notably influence alterations in the intake of these calorie-rich foods [39].

Adults with a higher BMI tend to exhibit greater FR, indicating a stronger reaction to food cues, emotional eating, and deriving pleasure from eating [39]. Today, they also struggle more with recognizing satiety cues and managing emotional eating, showing a complex relationship between psychological factors, eating behaviors, and weight [94]. Cross-cultural data show FR is linked to BMI and emotional eating across diverse populations, reinforcing its relevance in global nutritional research [95,96]. This link between higher BMI and increased FR persists today despite the associated negative outcomes [79].

Research after COVID shows that heightened FCR and reduced SR are associated with overeating and higher obesity rates [94]. These behaviors, recorded before COVID, existing on a spectrum, can be targeted to manage overeating and support weight loss [38]. Individuals more sensitive to food cues but less attuned to fullness are more prone to overeating and weight gain [11].

In the post-COVID period exposure to weight stigma increases brain responsiveness to high-calorie foods in people with overweight or obese, particularly in areas related to reward and sensory processing [40]. This heightened sensitivity may contribute to unhealthy eating behaviors and weight gain [39,79].

The behavioral susceptibility theory suggests that genetically influenced behaviors like FR interact with environmental factors, leading to overeating and weight gain [50]. The Regulation of Cues (ROC) intervention targets these behaviors by helping individuals with heightened FR better manage their responses to food cues and improve satiety regulations [11,51]. This approach highlights the combined impact of genetics and environment on eating behaviors and weight management [50].

In the post-COVID period, the Responsive Eating Pattern is characterized by high FR and is linked to food addiction (FA) and higher body weight [11,97,98]. Individuals with this pattern are highly reactive to food cues [94], gain significant pleasure from eating [99], eat quickly [100], struggle with recognizing fullness [101], eat in response to emotions [4], and are less selective about food choices [36]. These behaviors contribute to FA and can lead to weight gain [45,52], offering insights into how specific eating behaviors impact overall health [39,79].

**Table 2:** Papers included in this review are categorized under the theme ‘Food Responsiveness’ and its related sub-themes.

Eating Behavior Discourse	Sub-Domains	Paper Declaration Number
Food Responsiveness (FR)	Preference	[7,8,10,20,74,85,86,92,93,94,102,103,104,105,106,107]
	Desire	[30,38,46,71,73,94,108,109,110,111]
	Emotional State	[4,6,24,31,41,53,54,65,66,67,68,69,86,112,113,114,115,116]
	Overweight	[34,39,51,61,68,80,81,83,100,117]
	Body Mass Index	[9,18,35,39,51,61,64,68,79,80,81,82,83,98,105]

Today, the three subdimensions of hyperphagia—hedonic, homeostatic, and emotional—are positively correlated with increased FCR [79,112]. These excessive eating tendencies align with behaviors like emotional eating, heightened pleasure from food, increased sensitivity to food cues, and difficulty recognizing fullness [39,94]. This suggests that individuals with hyperphagia across these dimensions tend to eat in response to emotions, have higher FCR, and struggle with satiety recognition [39,63,101]. Table 2 the papers evaluated above for FR are presented while the flow chart 2 shows the influencing factors for FR leading to the proposition for the new post-COVID period.

#### 3.1.3. Emotional Overeating (EOE)

Emotional overeating refers to the tendency to consume food in response to negative emotions, such as stress, sadness, or anxiety, rather than hunger [64]. This behavior in the post-COVID-19 period is a central aspect of food approach behavior, where individuals turn to eating to cope with emotional distress [65,118]. Emotional distress post-COVID continues to drive overeating behaviors, especially in adolescents, highlighting the vulnerability of younger populations to stress-induced eating patterns [31,66,119]. Up-to-date research indicates that emotional overeating is associated with heightened sensitivity to food cures and a reduced capacity to recognize fullness, leading to weight gain and obesity [57,87,94]. This pattern is particularly problematic as it often leads to the consumption of calorie-dense, unhealthy foods [102,120].

The latest research findings have reaffirmed and expanded upon previous studies by He, Chen et al., indicating that individuals classified under the categories of EOE and EUE face the highest risks associated with depression, anxiety, stress, and impaired psychosocial functioning due to symptoms of disordered eating [112,113]. Additionally, these individuals exhibit lower levels of psychological flexibility [65,117].

Exploratory Factor analyses (EFA) revealed a single latent variable termed “Distress-Induced Overeating,” [103] which positively correlated with internal boredom proneness [121], tobacco use [64], attentional impulsiveness [41,73], susceptibility to emotional cues [122], and loss of control overeating [65]. Conversely, it showed a negative correlation with age and overall well-being [39,123,124].

Individuals who engage in EOE or EUE show today the highest levels of eating disorder symptoms and psychological distress [65,125]. Those who manage emotional fluctuations by overeating or undereating experience more severe symptoms of eating disorders and higher levels of anxiety, depression, and other negative emotions [67,112,123]. Satiety responsiveness moderates emotional eating, with cultural and age-specific variations, suggesting that interventions should be tailored to individual physiological and demographic profiles [126].

Individuals with binge eating disorder often show a strong link between negative emotions and excessive eating [127]. Stress, sadness, and anxiety frequently trigger overeating or binge eating as a coping mechanism [63,112]. This behavior temporarily alleviates emotional distress but can also reinforce and intensify the cycle of emotional eating, exacerbating the frequency and severity of episodes [65,122].

A significant positive correlation exists between EOE and EUE among students [9,125,128]. Nervousness and anger are key emotions linked to both behaviors [108]. Students may use EOE to cope with negative feelings or distract themselves, while EUE might result from stress-induced loss of appetite or attempts to control emotions [63,65].

A positive correlation exists between EOE and BMI, with higher EOE often linked to higher BMI [125]. Adults with a higher BMI tend to be more responsive to food cues and emotional triggers, leading to overeating [40], while showing reduced sensitivity to fullness and less mindful eating behaviors [39,80].

Control overeating positively correlates with prompting/encouragement to eat and negatively with instrumental feeding, which uses food as a reward or punishment [12]. This suggests that individuals who have more control over their eating are less influenced by such feeding practices and are more responsive to encouragement to eat, which can impact emotional feeding behaviors [8,12].

Today, individuals aged 60 to 80 score lower in food preferences like accessibility, convenience, sensory appeal, and comfort, as well as in food-related behaviors such as hunger response and EOE [48,74,75,101,129]. These studies found that older adults often experience moderate-to-severe seasonal variations in eating behaviors, with EOE being notably prevalent [58,130]. Concerns about weight partially mediate how these seasonal changes affect eating habits, highlighting the complex relationship between seasonal factors, weight concerns, and food behaviors in older adults [48,101,130].

Updated research shows that managing emotional eating, whether excessive or insufficient, can greatly improve quality of life, regardless of body measurements [112,123,131]. For adolescents, eating habits significantly impact overall well-being [81,82]. Additionally, past parental feeding practices are linked to current eating behaviors and weight status in young adults [37,104]. Obese participants scored significantly higher in FR and EOE compared to non-obese individuals, though there were no significant differences in hunger sensitivity [11,125]. This suggests that overweight and obese adults react more strongly to food cues and have reduced sensitivity to fullness [11,78]. Additionally, higher BMI is associated with increased EOE, and adolescents with obesity often show Sluggish Cognitive Tempo (SCT), which is linked to higher EOE [112,132]. Females exhibited elevated levels of EOE, whereas individuals classified as overweight or obese demonstrated heightened EOE and lower levels of EUE [53,109]. Despite their higher weight status, older adults reported lower levels of interoceptive attention, hunger drive, EOE, FCR, derived from eating compared to younger adults [48,58,101].

Emotional overeating (EOE) and food responsiveness (FR) were not significant predictors of changes in the consumption of high-energy-dense (HED) sweet and savory foods [11,39,79]. This suggests that changes in EOE and FR may not significantly affect the consumption of calorie-rich, sugary, or fatty foods [79,120]. Individuals with bulimia nervosa (BN) reported high levels of EOE [114,127]. During lab tasks, they showed increased desire to eat and a stronger eating response to high-calorie foods when experiencing negative emotions [41,64]. Additionally, a significant subgroup of these individuals may also experience food addiction (FA), even after accounting for binge eating disorder and BN [52,133].

**Table 3:** Papers included in this review categorized under the theme ‘Emotional Overeating’ and its related sub-themes.

Eating Behavior Discourse	Sub-Domains	Paper Declaration Number
Emotional Overeating (EOE)	Discomforts	[4,24,31,33,41,53,54,63,64,65,66,67,68,69,112,115,116,122,127,134]
	Obesity	[31,32,39,40,45,51,52,61,64,65,83,97,109,125,127,135,136]
	Body Mass Index	[32,34,35,39,40,45,51,61,62,64,68,79,80,81,82,83,98,123]
	Psychological Influence	[4,24,31,33,34,41,53,54,55,63,65,66,67,69,108,112,113,114,115,116,122,134,136,137]

The three dimensions of hyperphagia are positively linked to EOE and FR [11,120]. This indicates that individuals with higher levels of hyperphagia are more likely to engage in EOE and exhibit increased sensitivity to food cures and satiety signals [39,59]. The papers evaluated for EOE are presented in Table 1. Flow chart 3 shows the influencing factors for EOE which lead to the corresponding proposition for EOE in the new period.

#### 3.1.4. Enjoyment of Food (EF)

The enjoyment of food is defined as the positive emotional response and pleasure derived from eating, often associated with food approach behaviors such as increased responsiveness to food cues, cravings, and the desire to eat [94,99,118]. These behaviors are characterized by heightened interest in food, which can lead to overeating or difficulty resisting tempting foods [72]. Recent studies have shown that individuals who exhibit strong food approach behaviors tend to experience greater enjoyment of food, which is linked to increased intake of high-calorie, palatable foods [51,98,118]. Hedonic pleasure and food reward remain central to post-pandemic eating satisfaction, reinforcing the emotional and sensory dimensions of dietary gratification [32,138]. This relationship underscores the complexity of eating behaviors and their impact on dietary habits [79,139].

Recent research explores four areas related to food enjoyment: moral and ethical aspects, moderate eating for pleasure, intuitive and mindful eating, and the social benefits of communal dining [1,2,131]. Unlike excessive food intake, moderate eating enhances enjoyment, satisfaction, social interactions, and psychological well-being [112,121].

Another study highlighted four main themes showing how food affects quality of life (QOL): access and choices, preparation practices, health outcomes, and enjoyment [5,35,58,79,83]. These themes demonstrate food’s diverse impact on well-being and life satisfaction. EF, FR, and EOE did not significantly predict changes in the intake of HED foods, indicating that these factors have minimal influence on consuming calorie-rich foods [8,79,88].

Beliefs about the naturalness of food were negatively linked to health and enjoyment goals, suggesting that those who prioritize natural foods might value health benefits and enjoyment less [26,36]. Food safety beliefs had minimal impact on health or enjoyment goals, while taste beliefs influenced dietary variety but not enjoyment [1,35,140].

Preliminary findings showed race influenced EF, with participants of color reporting greater enjoyment than white participants [2,26]. Race influenced the link between EF and weight, while non-food enjoyment had little effect on weight [51,79,110].

Despite having higher body weight, older adults reported reduced levels of interoceptive, less pronounced H drive, lower incidence of EOE, diminished FCR, and decreased EF [40,58,94,101,125,132].

Greater EF, increased FCR, and frequent feelings of H significantly boost overall satisfaction with one’s food-related life. This means that those who experience more pleasure from eating and are more attuned to food cues tend to feel more satisfied with their food experiences. All preference scores were positively linked to EF, FR, and H, with the strongest correlations seen in preferences for dairy, snacks, and meat/fish [8,10]. This highlights the relationship between food preferences and behaviors related to food approach and avoidance [118]. Scores for food preferences were positively correlated with EF, H, and FCR, indicating that stronger food preferences are linked to these behaviors [8]. Conversely, more selective eaters tend to have lower food preference scores [141].

The connections observed were partially influenced by the current eating behaviors exhibited by young adults, including their EF, tendencies towards EOE, responsiveness to feelings of fullness, and pace of eating [39,100,112,135].

Age-related differences affect food choices, behaviors, and skills, revealing di-verse factors influencing dietary habits across age groups and genders [58,88]. Seasonal changes impact eating patterns in older adults, with weight concerns playing a role [58,75]. Higher BMI is associated with increased FR, EOE, and EF, along with decreased sensitivity to fullness [79]. Adolescents with obesity often show SCT, which correlates with greater EF and EOE [8,132]. The Responsive Eating Pattern, marked by high EF, FR, and emotional eating, links to food addiction and higher BMI [45,98].

The main barriers to adopting Restrictive Dietary Practices (RDPs) are EF and the desire for spontaneity and freedom in food choices [2,75]. These factors make it difficult for individuals to stick to restrictive diets, showing the challenge of balancing dietary goals with food pleasure and flexibility [35,112].

**Table 4:** Papers included in this review are categorized under the theme ‘Enjoyment of Food’ and its related sub-themes.

Eating Behavior Discourse	Sub-Domains	Paper Declaration Number
Enjoyment of Food (EF)	Predilection	[25,26,74,75,77,99,102,110]
	Like Eating, Satisfaction	[25,26,77,99,110,123,124,131,137]
	Body Mass Index	[35,36,45,51,61,64,68,79,80,81,83,100]
	Heaviness	[37,40,45,51,61,80,110,130,131]

Research shows that participants prefer enjoying food over focusing solely on functionality [26,110]. To promote healthier eating, interventions should focus on behavioral changes and improving nutritional knowledge [1,36]. Table 4 shows the domains and subdomains together with the evaluated papers for EF, while the flow chart 4 indicates the influencing factors for EF which lead to the proposition for the post-COVID period.

### 3.2. Food Avoidance Traits

#### 3.2.1. Satiety Responsiveness (SR)

Satiety Responsiveness (SR) refers to an individual’s ability to regulate food in-take in response to internal cues of fullness, influencing food approach behaviors [1,11,39]. High SR individuals tend to stop eating once they feel full, whereas low SR individuals may continue eating despite satiety, leading to overeating [60,101]. SR is a crucial factor in under-standing eating patterns and obesity risk [50,79]. Recent studies reaffirm SR’s predictive role in weight regulation and its interaction with emotional eating and food cue reactivity [142]. Research over the last decade highlights the role of genetic, environmental, and psychological factors in shaping SR, emphasizing its importance in the development of interventions for weight management and healthy eating habits [39,50].

Sensitivity to food, EF, EOE, and responsiveness to fullness had minimal impact on the intake of HED foods [11,39]. The study also found limited evidence connecting SR to eating speed, which aligns with findings that individual differences in eating rate play a role in body weight and glycemia control [100]. A 2024 meta-analysis confirmed that slower eating and higher SR are associated with improved glycemic profiles and reduced BMI in adults [143]. Individuals with low satiety sensitivity are more likely to overeat due to stronger hunger signals and food cravings, making them more susceptible to excessive eating, consistent with theories of behavioral susceptibility to obesity [39,50].

Assessing hunger, fullness, and their link to appetite-regulating peptides offers insights into obesity [57]. Women had higher EOE and greater SR [53,68,69]. Obese participants scored lower in SR and EUE [39,101]. No differences were observed in EF or H [27]. This suggests that overweight and obese adults are more reactive to food cues and less sensitive to fullness compared to those with normal weight [51,79]. Recent findings from a 2025 cross-sectional study in Portugal showed that SR scores were significantly lower in obese adults, especially those with high emotional eating traits [144].

Studies show that increased FCR and reduced sensitivity to satiety are linked to overeating and overweight or obesity [10,79]. Emerging evidence suggests that FCR and SR exist on a continuum, indicating that addressing these factors could aid in managing overeating and promoting weight loss [101,136]. A 2025 behavioral intervention trial demonstrated that enhancing SR through mindfulness and cue exposure therapy led to significant reductions in binge eating and BMI over 12 weeks [145].

In midlife women, lower sensitivity to hunger and satiety signals was associated with higher stress and body fat [67,101,131]. While most eating behaviors correlated positively with food-related satisfaction, SR had a negative correlation, highlighting gender differences in responses to fullness [39,69].

Our study found that sensitivity to internal satiation cues (SISC) correlates with satiety responsiveness (SR) and affects decisions to stop eating [39]. The ROC intervention aims to modify responses to food cues and fullness to address overeating and weight gain [51,94,110]. The Responsive Eating Pattern, marked by low SR, and selective preferences, is linked to food addiction (FA) and higher BMI [45,98]. Decreased SR and faster eating are associated with elevated BMI, with higher BMI individuals showing greater food approach behaviors and reduced SR [100].

The findings suggest that individuals with low SR are more prone to overeating due to heightened sensitivity to hunger and food cravings, as they struggle to recognize and respond to fullness [39,101]. This aligns with recent neurobehavioral models showing that impaired interoceptive awareness and reward sensitivity jointly predict low SR and compulsive eating behaviors [111].

**Table 5:** Papers included in this review are categorized under the theme ‘Satiety Responsiveness’ and its related sub-themes.

Eating Behavior Discourse	Sub-Domain	Paper Declaration Number
Satiety Responsiveness (SR)	Food Graving	[27,39,41,60,61,73,87,94,114,120]
	Pathogenesis of Obesity	[11,39,51,57,60,61,62,78,79,98,133]
	Anxiety Symptoms, Stress, Stimuli	[24,31,34,41,53,63,64,65,66,67,68,69,70,112,115]
	Food Addiction, Pleasure	[34,38,45,46,52,61,76,97,98,109,120,125,133,136]
	Body Mass Index	[35,39,45,51,61,64,68,76,79,80,81,82,83,98]

Understanding satiation and satiety can inform the development of future food products, as these physiological processes are central to effective dietary interventions [61,78]. Low SR is associated with reduced awareness of fullness and increased overeating risk, indicating variations in how people recognize and respond to satisfaction signals [10,39]. In males, better cooking skills and higher SR were linked to increased consumption of highly processed foods (HPF) [36,88]. In females, older age and greater safety/nutrition knowledge were associated with lower HPF intake, while higher SR correlated with increased HPF consumption [36,88,102]. The papers evaluated above for SR are presented in Table 5. Flow chart 1 shows the influencing factors for FR which lead to the proposition for the new era.

#### 3.2.2. Emotional Under Eating (EUE)

Emotional Under-Eating (EUE) refers to a behavior where individuals reduce their food intake in response to emotional stress, rather than increasing it [108]. This response is characterized by a diminished appetite and lower food consumption when experiencing negative emotions [58,120]. EUE contrasts with EOE, where stress leads to increased eating [63]. Recent meta-analytic findings confirm that EUE is significantly associated with emotion regulation difficulties and internalizing symptoms such as anxiety and depression, particularly in adolescents and young adults [115]. Recent studies highlight EUE’s role in food approach behaviors, showing its association with heightened stress and emotional regulation difficulties [76,118].

The study found that control behaviors such as EUE, EOE, SE, significantly improve adolescents’ quality of life, regardless of their body metrics [117,123]. Healthier eating habits enhance both physical and mental well-being [1]. A 2025 study from the UB-EATS cohort found that adolescents experiencing food insecurity and perceived stress were more likely to exhibit disordered eating patterns, including EUE, especially when parental stress was also elevated [146].

The study found that many older adults experienced significant seasonal changes in their eating patterns, with moderate levels of EUE, EOE, and EF [75,130]. Adults with higher BMI showed lower SR and EUE [11,39,101]. Women had higher EOE and SR, while those with overweight or obesity had increased EOE and decreased EUE [53,68]. A multicenter study in Brazil found that college-aged women with high stress levels were more likely to show EUE than EOE, suggesting cultural and gender-specific coping mechanisms [116]. Gender and weight status significantly influence eating behaviors [88,105]. For adolescents, positive food preferences are associated with hunger responsiveness, EF, and EUE, and being less selective about food [86,93,106]. However, EUE, EOE, and FR do not notably impact the consumption of HED foods [73,120]. A 2025 cross-sectional study in Saudi Arabia confirmed that perceived stress was negatively correlated with appetite and food intake among female university students, reinforcing the role of EUE in stress-related eating suppression [147].

**Table 6:** Papers included in this review categorized under the theme ‘Emotional Under Eating’ and its related sub-themes.

Eating Behavior Discourse	Sub-Domains	Paper Declaration Number
Emotional Under Eating (EUE)	Inclination	[6,24,53,54,112,113,114,115,116,121,134,146]
	Negative Emotions	[31,33,53,54,63,65,66,67,69,112,113,114,115,116,134]

The findings confirm and expand on He, Chen et al., showing that EUE and EOE are strongly linked to depression, anxiety, and psychosocial impairment, alongside lower psychological flexibility [67,112,113]. These patterns reveal how food preferences are influenced by tendencies to approach or avoid food, offering deeper insights into eating behaviors [106,118]. In Table 6, the papers evaluated above for EUE are presented, and the flow chart 6 below shows the influencing factors for hunger which led to the proposition for the respective parameter for the post-COVID period.

#### 3.2.3. Food Fussiness (FF)

Food fussiness, a subcategory of food approach behavior, refers to the tendency of individuals, particularly children, to exhibit selective eating patterns, often rejecting unfamiliar or disliked foods [15,107]. This behavior is characterized by a limited range of accepted foods and reluctance to try new items, which can impact dietary variety and nutritional intake [15,16,101]. Recent twin studies confirm that FF is a stable trait from toddlerhood to adolescence, with genetic factors playing a dominant role in its persistence [148]. Studies over the last decade highlight the role of parental influence, sensory sensitivity, and genetic predisposition in shaping FF [12].

FF is related to behaviors such as H, FR, and EF [11]. These findings indicate that FF is connected to distinct patterns of food approach and avoidance, influenced by individual food preferences [1,8,79]. A 2024 systematic review found consistent associations between FF and lower dietary diversity, especially in children with heightened sensory sensitivity and anxiety traits [149].

The study found that many older adults experienced moderate to severe FF alongside fluctuations in their eating patterns throughout the year [48,75,130]. Weight concerns partially influenced these changes, highlighting the complex relationship between psychological factors and variations in eating habits [64,130]. An integrative review published in 2025 emphasized the role of family dynamics and caregiver feeding practices in shaping FF across the lifespan, especially in older adults with cognitive decline or mood disorders [150].

FF negatively correlated with preference scores, meaning those who are more selective about food tend to have lower preference scores [139,151]. In contrast, behaviors like H, FR, and EF positively influenced preferences [11]. These findings suggest that while a strong desire for food enhances preferences, being overly selective diminishes them [85,141].

Women scored higher in H, FR, EF, and EUE than men, but they did not show higher levels of FF [53,68,118,128]. This indicates that while women generally have a stronger reaction to food-related factors, their degree of food selectivity is comparable to that of men. A 2025 cross-cultural study found that gender differences in FF were minimal, but women exhibited greater emotional responsiveness to food cues, which influenced their food variety and intake [152].

In both men and women, higher Healthy Eating Index-2015 (HEI-2015) scores were associated with healthier food choices and lower FF [1,102]. Men with higher HEI-2015 scores preferred organic foods and were less picky about their choices [26,105]. Similarly, women with higher HEI-2015 scores made health-focused food choices and were also less particular about their food [105,131].

FF, SR, and SE were all negatively correlated with BMI [39,100,151]. This suggests that individuals who feel full more easily eat at a slower pace and are more particular about their food choices generally have a lower BMI. Recent findings from the Gemini twin cohort reinforce that FF is inversely related to BMI and positively associated with slower eating and heightened satiety sensitivity [153].

**Table 7:** Papers included in this review categorized under the theme ‘Food Fussiness’ and its related sub-themes.

Eating Behavior Discourse	Sub-Domains	Paper Declaration Number
Food Fussiness (FF)	Tension	[4,31,53,54,63,68,74,121,139,141,150]
	Food Preference, Sensory Sensitivity	[5,35,36,74,75,92,93,95,96,139,141,148,149,151,152,153]

After adjusting for age and gender, higher Generalized Anxiety Disorder-7 (GAD-7) scores, which assess the severity of generalized anxiety symptoms, were significantly associated with increased H, EOE, heightened FCR, and greater FF [52,70,94,107,112]. This suggests that individuals with higher anxiety levels experience more H, are more prone to emotional eating, respond more strongly to food, and are more particular about their food. Conversely, higher GAD-7 scores were linked to lower EF, indicating that increased anxiety is associated with reduced pleasure from eating [79].

#### 3.2.4. Slowness in Eating (SE)

Slowness in eating, an important element of food approach behavior, describes the intentional pace at which people eat their meals [77,118]. This practice, involving a longer eating duration, can affect feelings of fullness and total calorie intake [100,101]. Research indicates that eating slowly relates to greater satiety, lower calorie intake, and improved weight control [61,100]. A 2025 study published in The Lancet Child & Adolescent Health confirmed that slower eating and greater satiety sensitivity in early childhood were associated with lower risk of eating disorder symptoms in adolescence [62]. It is also associated with mindfulness during meals, enhancing awareness of H and fullness cues, and influencing eating behaviors [11,131]. Recent findings from Frontiers in Psychology (2025) show that mindful eating practices significantly enhance self-regulation and reduce impulsive food intake, reinforcing the role of SE in healthy eating behavior [137].

The study demonstrated that control behaviors like SE, EOE, and EUE can substantially improve quality of life, regardless of body measurements [39,122,123]. Since dietary behaviors are central to adolescents’ lifestyles, they are vital for enhancing overall well-being [92]. A 2025 meta-analysis in the Journal of Eating Disorders found that emotion regulation difficulties are closely linked to disordered eating and BMI, suggesting that improving EOE and EUE may enhance psychological well-being and dietary control [134]. Addressing these eating patterns, particularly SE, can lead to significant improvements in quality of life, underscoring the importance of focusing on dietary habits for better well-being [39,117,123].

Compared to younger age groups, individuals aged 60–80 had lower scores in areas like accessibility and EF but valued tradition, organic options, and safety more [26,48]. Notably, they ate more slowly than younger groups, reflecting distinct food priorities and eating behaviors aligned with their lifestyle changes [58,100]. A 2024 study in the Journal of Sports Science found that older adults exhibited higher SE and lower food responsiveness, aligning with their preference for mindful and traditional eating patterns.

SE was associated with lower BMI, indicating that individuals who eat more slowly typically have lower body weights [100,101]. Conversely, EOE was linked to higher BMI, suggesting that people who eat in response to emotions tend to have higher body weights [112,125]. Both SE and SR were related to reduced BMI, while EOE was associated with increased BMI, highlighting the intricate connection between eating habits and body weight [39]. Recent evidence from a systematic review in Current Nutrition Reports (2024) supports these associations, showing that SE and SR are protective against obesity, while EOE increases risk. Adults with higher BMIs scored lower on “food avoidance” behaviors SR and SE. This indicates that those with higher BMIs are less sensitive to fullness cues, less likely to eat slowly, and less prone to emotional undereating [10,51].

Obese individuals had lower scores for SE, SR, and EUE [11,39]. They also had notably higher scores for FR and EOE compared to those who were not obese [112,118,125]. This indicates that overweight and obese adults are more reactive to food cues and less sensitive to feelings of fullness, contributing to higher body weight [50,94].

Women showed higher EOE and SR than men [53,54,68]. In contrast, overweight and obese individuals had higher EOE but lower EUE and SE [39,51,60]. This indicates that while women are more attuned to fullness, overweight or obese individuals often eat more quickly and are less sensitive to satiety cues, contributing to higher body weight [100,101].

**Table 8:** Papers included in this review categorized under the theme ‘Slowness in Eating’ and its Related sub-themes.

Eating Behavior Discourse	Sub-Domains	Paper Declaration Number
Slowness in Eating (SE)	Eating Rate	[5,35,56,60,61,62,74,77,87,100,101,103,130,137]

The relationships were partly influenced by the eating characteristics of young adults, such as EF, EOE, SR, and SE [88]. These behaviors affected how different factors influenced eating behaviors and dietary patterns [1,2,5]. For example, those who enjoy food more or are prone to EOE may experience different impacts on their eating habits compared to those who are more responsive to fullness or eat more slowly [36,100]. This mediation highlights the complex interplay between psychological and physiological factors in shaping eating behaviors [61,94]. The papers evaluated above for SE are presented in Table 8. Flow chart 8 shows the influencing factors for hunger which lead to the proposition for SE for the post-COVID period.

### 3.3. Conceptual Mapping of Eating Behavior Parameters in the Post-COVID Era

This section presents a structured overview of the interrelationships among eight key eating behavior parameters: Hunger (H), Food Responsiveness (FR), Emotional Overeating (EOE), Enjoyment of Food (EF), Satiety Responsiveness (SR), Emotional Undereating (EUE), Food Fussiness (FF), and Slowness in Eating (SE). Based on the literature reviewed, a conceptual correlation table (Table 9) was developed to describe the nature of each relationship—whether positive, inverse, or neutral—without relying on statistical metrics. This qualitative mapping highlights how emotional and physiological factors interact to shape sustainable food behaviors in the post-pandemic context.

**Table 9:** Conceptual correlation table of eating behavior parameters.

Parameter	Related Parameters	Nature of Relationship
Hunger (H)	FR, EOE, EF	Positively linked—hunger increases food responsiveness, emotional overeating, and enjoyment of food
	SR, SE	Inversely linked—hunger decreases satiety responsiveness and slowness in eating
Food Responsiveness (FR)	H, EOE, EF	Positively linked—responsive individuals tend to feel hungrier, overeat emotionally, and enjoy food more
	SR, SE	Inversely linked—responsiveness reduces satiety signals and slows eating pace
Emotional Overeating (EOE)	H, FR	Positively linked—emotional overeating rises with hunger and food responsiveness
Enjoyment of Food (EF)	H, FR	Positively linked—enjoyment increases with hunger and responsiveness
	FF	Inversely linked—picky eaters enjoy food less
Satiety Responsiveness (SR)	SE	Positively linked—those who feel full easily tend to eat more slowly
	H, FR	Inversely linked—hunger and responsiveness reduce satiety awareness
Emotional Undereating (EUE)	FF, SE	Positively linked—emotional undereaters are often picky and eat slowly
Food Fussiness (FF)	EUE	Positively linked—picky eaters often eat less when emotional
	EF	Inversely linked—fussiness reduces food enjoyment
Slowness in Eating (SE)	SR, EUE	Positively linked—slow eaters are more responsive to fullness and prone to emotional undereating
	H, FR	Inversely linked—hunger and responsiveness speed up eating

**Figure 10:**
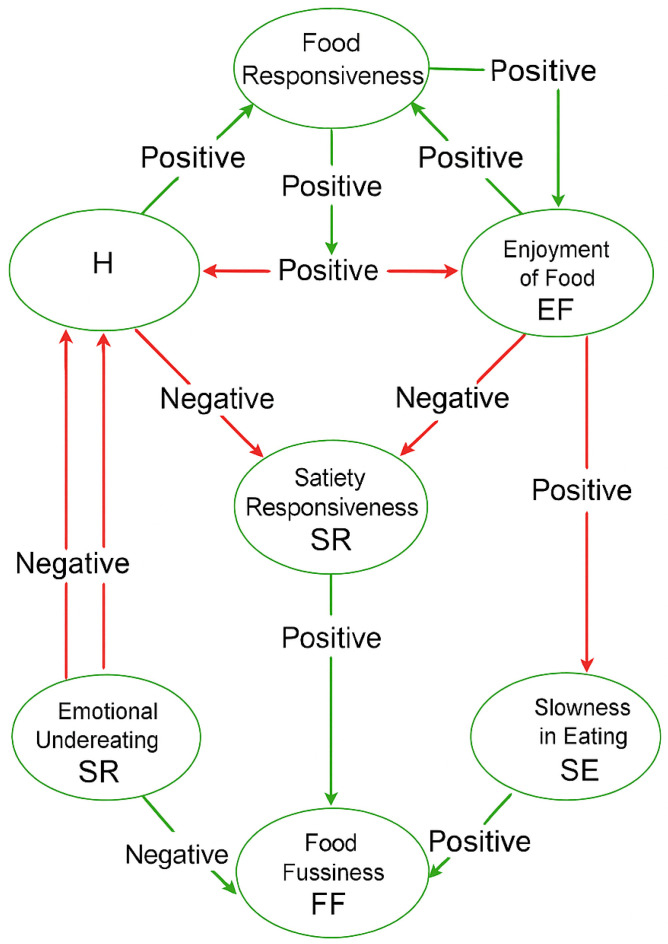
Flow Chart 9—Consumers’ Eating Behavior Index. Conceptual flow chart integrating all eight parameters into a holistic framework for understanding post-COVID eating behavior.

To visually support these findings, a flow chart (Flow Chart 9.) diagram illustrates the directional links between parameters using color-coded arrows (Figure 10). Green arrows represent positive associations (e.g., Hunger increases Food Responsiveness), while red arrows indicate inverse relationships (e.g., Hunger reduces Satiety Responsiveness). Together, the table and diagram offer a comprehensive framework for understanding the behavioral dynamics that influence food choices and consumption patterns, providing a valuable tool for researchers, clinicians, and policymakers aiming to promote healthier and more sustainable eating habits.

## 4. Conclusions

Our study highlights the complex nature of eating behaviors in the post-COVID period, and the changes recorded so far, illustrating how psychological factors, age, gender, and personal characteristics converge to influence individuals’ responses to food cues and their dietary management. We emphasize the intricate interplay between these elements, revealing how emotional states, life stages, and personal traits collectively shape eating patterns. By examining these interconnections, based on the 8 specific propositions stated above, our research provides a comprehensive understanding of the diverse factors that impact food-related behaviors today, stressing the importance of considering psychological and demographic variables in addressing dietary habits and developing effective interventions. This multifaceted approach offers valuable insights into how different factors contribute to the way people approach food and manage their nutrition.

The COVID-19 pandemic has had a profound impact on eating behaviors, bringing to light the complex interplay between emotional and physiological factors in shaping dietary choices and overall nutrition. During this period, heightened stress, anxiety, and uncertainty have led to increased emotional eating, altered food preferences, and changes in eating patterns, underscoring the critical connection between mental health and eating behavior.

Increased hunger during the pandemic has been shown to enhance food enjoyment, but it also exacerbates cravings. Emotional states, including stress and happiness, significantly influence the intensity and nature of food cravings, illustrating a complex interaction between physiological hunger and emotional factors. This inter-play affects how individuals experience and respond to their hunger cues and food-related emotions.

Older adults exhibit distinct food preferences and eating behaviors compared to younger individuals. They often place higher value on tradition and food safety and show lower levels of emotional eating and food responsiveness. This demographic’s eating behaviors are shaped by different priorities and experiences, which contrast with the eating patterns observed in younger populations.

Hedonic hunger, driven more by the pleasure of eating than by physiological need, is closely linked to higher BMI and obesity. This type of hunger, which is more prevalent among women and tends to decrease with age, contributes significantly to the consumption of high-calorie, sugary foods. This pattern of eating for pleasure rather than necessity has been associated with weight gain and increased risk of obesity.

High food responsiveness, while associated with greater enjoyment of food, also leads to overeating and obesity, particularly when combined with diminished satiety signals. Individuals with high food responsiveness may struggle to regulate their food intake effectively, leading to increased caloric consumption and potential weight gain.

Psychological distress, such as anxiety and depression, has been found to exacerbate emotional overeating and alter eating patterns, potentially leading to unhealthy eating behaviors and weight gain. These emotional states disrupt normal eating patterns and contribute to difficulties in managing dietary habits.

Gender differences further complicate the relationship between emotional eating and satiety responsiveness. Women generally exhibit higher levels of emotional eating and satiety responsiveness compared to men, leading to different eating behaviors and weight outcomes. This suggests that gender plays a significant role in influencing how individuals respond to food cues and manage their eating behaviors.

The current review, drawing up the findings discussed, presents eight specific suggestions for each of the eight key parameters that define appetitive traits. These suggestions are intended to serve as both practical and theoretical foundations for creating a new “Consumers’ Eating Behavior Index,” which can be utilized in future research and applications.

- Develop metrics that assess the interaction between emotional states and physiological hunger cues to provide a comprehensive understanding of eating behaviors.
- Create age-specific profiles to account for differences in food preferences, emotional eating, and satiety responsiveness, enhancing targeted interventions for various age groups.
- Include measures for hedonic hunger to evaluate the influence of pleasure-driven eating on BMI and obesity, particularly focusing on gender differences.
- Develop tools to measure sensitivity to food cues and satiety signals, identifying individuals at risk for overeating and obesity.
- Incorporate assessments of psychological distress, including anxiety and depression, to understand their effects on eating patterns and food preferences.
- Address gender-specific and weight-related differences in emotional eating and food responsiveness to tailor interventions more effectively.
- Evaluate behavioral patterns such as eating speed and food responsiveness, linking them to overall eating satisfaction and health outcomes.
- Implement longitudinal studies to track changes in eating behavior over time, especially in response to significant life events or stressors, to better predict and manage long-term dietary habits.

The findings underscore the importance of developing targeted public health interventions and policies to address these evolving dietary challenges and promote overall sustainable well-being in the post-pandemic index. The “Consumers’ Eating Behavior Index” offers a practical tool to guide and support these strategies and objectives. Overall, these efforts will contribute to the field of sustainable nutritional science and behavioral research.

Despite the conclusions drawn from the current review, additional research will be essential in the coming years to confirm their accuracy and reliability. While the findings provide valuable insights into the interplay between emotional, physiological, and behavioral factors influencing eating habits, further studies are necessary to validate these insights across diverse populations and contexts.

This ongoing research will help refine the proposed concepts and ensure that they accurately reflect and predict consumer eating behaviors. Continued investigation will also be crucial for adapting the proposed framework to evolving trends and emerging issues in nutrition and psychology. By systematically addressing these research gaps, future studies can bolster the credibility of the new “Consumers’ Eating Behavior Index” and enhance its utility for both theoretical exploration and practical application in understanding and managing eating behaviors.

## Data Availability

Not applicable.
